# The Pathogenic Diagnosis in Pediatric Diabetology: Next Generation Sequencing and Precision Therapy

**DOI:** 10.3390/medicina59020310

**Published:** 2023-02-08

**Authors:** Giovanna Maione, Fernanda Iafusco, Angela Zanfardino, Alessia Piscopo, Gulsum Ozen, Dario Iafusco, Nadia Tinto

**Affiliations:** 1 Department of Molecular Medicine and Medical Biotechnology, University of Naples “Federico II”, Via S. Pansini, 5-80131 Naples, Italy; 2 CEINGE Advanced Biotechnology, Via G. Salvatore, 486-80145 Naples, Italy; 3 Regional Centre of Paediatric Diabetology “G. Stoppoloni”, Department of Woman, Child and of General and Specialized Surgery, University of Campania “L. Vanvitelli”, Via L. De Crecchio, 2-80138 Naples, Italy; 4 Department of Pediatrics, University of Health Science, Kecioren Training and Research Hospital, Ankara 06100, Turkey

**Keywords:** monogenic diabetes, precision diabetology, next-generation sequencing

## Abstract

In pediatric diabetology, a precise diagnosis is very important because it allows early and correct clinical management of the patient. Monogenic diabetes (MD), which accounts for 1–6% of all pediatric–adolescent diabetes cases, is the most relevant example of precision medicine. The definitive diagnosis of MD, possible only by genetic testing, allows us to direct patients to more appropriate therapy in relation to the identified mutation. In some cases, MD patients can avoid insulin and be treated with oral hypoglycemic drugs with a perceptible impact on both the quality of life and the healthcare costs. However, the genetic and phenotypic heterogeneity of MD and the overlapping clinical characteristics between different forms, can complicate the diagnostic process. In recent years, the development of Next-Generation Sequencing (NGS) methodology, which allows the simultaneous analysis of multiple genes, has revolutionized molecular diagnostics, becoming the cornerstone of MD precision diagnosis. We report two cases of patients with clinical suspects of MD in which a genetic test was carried out, using a NGS multigenic panel, and it clarified the correct pathogenesis of diabetes, allowing us to better manage the disease both in probands and other affected family members.

## 1. Introduction

The conceptual wall that defined pediatric-onset diabetes as “type 1”, “autoimmune”, “insulin-dependent” and adult-onset diabetes as “type 2”, “non-autoimmune”, “non-insulin-dependent” is, like many other walls, finally “collapsing” thanks to the unavoidable attempt to define the exact pathogenesis of each new case [1]. 

If the first diagnostic step is the autoimmune markers of diabetes (GAD, IA2, IAA, and ZnT8 antibodies) assay, and these are absent, the entire diagnostic process of non-autoimmune diabetes mellitus is opened. 

We can paradoxically report that the major discovery of the 21st century in pediatric diabetology is the great pathogenic heterogeneity of diabetes mellitus, which is not only due to the autoimmune, TH1-mediated, destruction of pancreatic beta cells, but may be due to the involvement of numerous genes related to insulin secretion or to mechanisms of insulin resistance. Monogenic diabetes (MD) comprises a heterogeneous group of disorders caused by mutations in a single gene. It accounts for a relatively small proportion of diabetes cases, ranging from 1% to 6% of the diabetic pediatric and young adult population; however, its real prevalence is still underestimated. This pathology represents an excellent example of precision medicine, as once the genetic defect is identified, targeted therapy can be practiced, which makes it possible, in most cases, to avoid insulin therapy and attempt alternative therapies such as oral hypoglycemic agents (Precision Pediatric Diabetology) that have a significant impact on the quality of life of patients and healthcare costs [2,3]. Unfortunately, the genetic and phenotypic heterogeneity of MD can make it difficult to identify the specific form, preventing it from reaching a definitive diagnosis. 

In the last few years, the development of advanced sequencing methods, such as Next-Generation Sequencing (NGS), which allows the simultaneous analysis of multiple genes, has led us to reach a diagnosis rate of MD higher than that obtained in the past. In a recent paper, the authors reported that NGS is able to identify MD in 16% of patients who develop non-autoimmune diabetes in late adolescence/young adults [4]. As in many other fields of medicine, such as MD, NGS techniques have become the cornerstone of precision diagnosis. We have moved from the need to test gene by gene by Sanger sequencing on the bases of clinical suspicion and clinical phenotype to the possibility of performing multigene panel analyses, allowing us to diagnose rare monogenic diabetes subtypes previously underdiagnosed or even undiagnosed [5,6,7]. The multigenic approach is proving to be very practical and capable of providing results, often disengaging from clinical diagnosis. Furthermore, this methodology may also allow to identify digenic mutations that would otherwise remain undetected in a monogenic approach [7,8].

Herein, we report the clinical impact of the application of NGS in two patients with onset diabetes mellitus in childhood followed at the Regional Center of Pediatric Diabetology G. Stoppoloni of the University of Campania Luigi Vanvitelli. 

## 2. Materials and Methods

Peripheral blood samples were collected after obtaining signed informed consent from both patients and their relatives.

Genomic DNA was extracted from leukocytes with a QIAamp DNA Blood Mini Kit (Qiagen, Hilden, Germany), according to the manufacturer’s instructions. A NGS panel, including 42 genes associated with non-autoimmune diabetes, was used to analyze the probands. For each gene, the coding regions, 50 bp in each of the intronic boundaries, the promoter, and the 3′ UTR, for a total target size of about 1 Mb, were analyzed. A total of 50 ng of gDNA was processed through the SureSelectQXT Target Enrichment system (Agilent Technologies, Santa Clara, CA, USA) for Illumina multiplexed sequencing. Sequencing reactions were performed on the MiSeq instrument (Illumina, San Diego, CA, USA). The sequence reads were aligned to the human reference genome (hg38) using the Alissa Align & Call v1.0.2.10 tool (Agilent Technologies, Santa Clara, CA, USA). The detected variants were evaluated by Alissa Interpret v5.2.6 CE IVD software (Agilent Technologies, Santa Clara, CA, USA), using GRCh38.p2 and several databases of genomic variants. To confirm the identified variants in probands and their relatives, standard Sanger sequencing was performed. 

## 3. Case Report

### 3.1. Case 1

**Figure 1:**
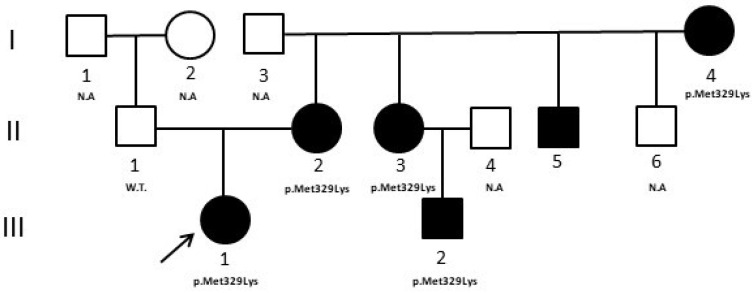
Case 1 pedigree. The pedigree shows the segregation of the *HNF4A* variant detected in the proband. Filled symbols indicate subjects with diabetes. W.T. = Wild type. N.A. = not analyzed. ↗ = proband.

We report the case of a female patient, the only child of non-consanguineous parents, age of 11 years for ketoacidosis and hyperglycemia. She was treated with insulin at the dose of 0.8 U/Kg/day in four administrations daily. After 3 months, the insulin was reduced to 0.2 U/Kg/day and then completely suspended with HbA1c values in target. Her autoimmune markers of diabetes (GAD, IAA, IA2, and ZnT8) were negative and a positive family history of diabetes (mother, mother’s siblings, and maternal grandmother) was present. A few years earlier, the diabetic patient’s cousin, with the same phenotype, was analyzed by Sanger sequencing of the *HNF1A* gene and no significant variants were revealed, but only multiple homozygous polymorphisms that allowed us to hypothesize the presence of a large gene deletion in this gene. Based on these results, a gene dosage analysis with a multiplex ligation probe assay (MLPA) (MRC–Holland kit, Amsterdam, Holland) which provides the simultaneous analysis of *HNF4A*, *GCK*, *HNF1A*, and *HNF1B* genes, was performed, but no deletions were detected. When the patient came to our attention, to characterize the pathogenesis of diabetes in this family, an NGS analysis was performed that revealed the variant c.986T > A (p.Met329Lys) in exon 8 of the *HNF4A* gene, which was then also confirmed with standard Sanger sequencing. The variant, not reported in the Human Gene Mutation Database (HGMD professional 2022.3) or in any known genomic variants database, was classified, according to the American College of Medical Genetics and Genomics and the Association for Molecular Pathology (ACMG/AMP) classification criteria and ClinGen Monogenic Diabetes Variant Curation Expert Panel (MDEP, https://clinicalgenome.org/affiliation/50016/, accessed on 1 February 2023) criteria, as “likely pathogenic”, because: (A) it is absent in population databases (absent from gnomAD, Exome sequencing project, 1000 genome project) (PM2_supporting); (B) it is located in a mutational hotspot and/or critical and well-established functional domain (PM1_supporting); (C) multiple lines of computational evidence suggest a deleterious effect on the gene or gene product (PP3_moderate); (D) it co-segregates with the disease in multiple affected family members in a gene definitively known to cause the disease (PP1_strong) [8]. Moreover, bioinformatics predictions by the Alamut software [(http://www.interactive-biosofware.com/alamut-visual/) Alamut Visual plus v1.5.1, accessed on 17 January 2023] report that the variant is located in a moderately conserved nucleotide among species, and the amino acid Lysine has physicochemical differences with respect to the wild-type Methionine, indicating its functional relevance [9]. The molecular analysis extended to relatives showed that the variant was present in all diabetic relatives (I:4, II:2, II:3, II:5, III:2) and absent in the normoglycemic father (II:1) (Figure 1).

### 3.2. Case 2 

**Figure 2:**
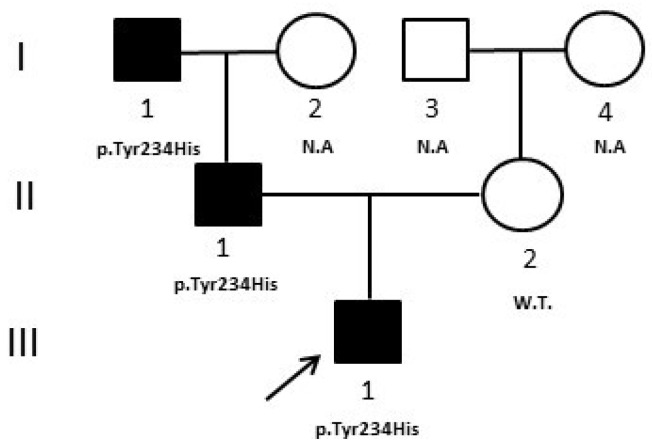
Case 2 pedigree. The pedigree shows the segregation of the *GCK* variant detected in the proband. Filled symbols indicate subjects with diabetes. W.T. = Wild type. N.A. = not analyzed. ↗ = proband.

We report the case of a male patient, the only child of non-consanguineous parents, who came to our observation at 3 years of age for fasting hyperglycemia with a Hb1Ac value of 6%. Immediately after birth, the patient had surgery for esophageal atresia and renal malformations were diagnosed. During the follow up, at the age of six months, mild hyperglycemia (129 mg/dL) was discovered. He was negative for the autoantibodies characteristic of autoimmune forms of diabetes and had a positive family history of diabetes (father and paternal grandfather). Due to the presence of extra-pancreatic malformations, clinicians directed the patient to *HNF1B* gene analysis [10]. Sanger sequencing was negative, therefore, since large or whole gene deletions can be responsible for up to 40% in HNF1B-MODY, a gene dosage analysis with MLPA (MRC—Holland kit, Amsterdam, Holland) was performed; however, no deletion/duplication was identified. Based on these previous results, we decided to perform a multigene panel analysis by NGS that evidenced a heterozygous variant in exon 7 of the Glucokinase (*GCK*) gene (c.700T > C-p.Tyr234His), also confirmed by Sanger sequencing. The variant is reported in HGMD (CM096864) and, according to ACMG/AMP classification and ClinGen/MDEP criteria, was classified as “likely pathogenic” because: (A) it is absent in population databases (absent from gnomAD, Exome sequencing project, 1000 genome project) (PM2_supporting); (B) multiple lines of computational evidence suggest a deleterious effect on the gene or gene product (PP3_strong); (C) missense variant in a gene that has a low rate of benign missense variation and in which missense variants are a common mechanism of disease (PP2_supporting) [8]. Moreover, bioinformatics predictions by Alamut software [(http://www.interactive-biosofware.com/alamut-visual/) Alamut Visual plus v1.5.1, accessed on 17 January 2023] report that the variant is located in a highly conserved nucleotide among species, indicating its functional relevance. The molecular analysis of relatives showed that the variant was present in the father (II:1) and in the paternal grandfather (I:1), who both have diabetes, and absent in the normoglycemic mother (II:2) (Figure 2).

## 4. Discussion

The genetic and clinical heterogeneity of the different forms of MD imposes the need to refer to experts in the field of this disease who can take advantage of the most advanced methodology, such as NGS, to analyze multiple genes simultaneously, in an effort to reach the maximum genetic yield [11]. In the last few years, the extensive use of NGS has permitted considerable progress in MD molecular diagnostics leading to an increased frequency of diagnoses [12,13]. Molecular diagnosis has a relevant role to confirm the specific subtype of MD, allowing us to predict the clinical course of the disease and identify the appropriate treatment, including during pregnancy, to lead to an improvement in the quality of life for affected patients and their familiars [2,12,14,15]. 

In the present study, we described two patients referred to in our laboratory with a clinical suspicion of MD, previously analyzed by Sanger sequencing for a single gene, and analyzed them with a NGS panel including 42 genes. In both patients, a gene variant was identified in genes different from the initial clinical suspicion.

In case 1, the NGS result induced clinicians to revise the clinical characteristics of all subjects of the family. In particular, it was evidenced that all subjects positive for the variant (Figure 1, II:2, II:3, II:5) were macrosomic at birth, and developed neonatal hypoglycemia, frequent features of HNF4A-MODY1, that at first were not considered. The mother’s non-diabetic brother (Figure 1, II:6), not analyzed, developed severe neonatal hypoglycemia at birth that determined a serious motor deficit in adulthood. HNF4A-MODY1 is characterized by a greater number of beta pancreatic cells at birth; therefore, fetuses and newborns with an *HNF4A* mutation have excessive insulin secretion that determines a marked increase in birth weight (~800 g) and a very high risk of macrosomia and hypoglycemic crises. Later, with growth, there is a progressive reduction of beta-cell function that leads to the onset of diabetes at puberty or in young adults. 

Thanks to NGS, our patient received a precise diagnosis and was able to switch the therapy from insulin to oral hypoglycemic agents, sulfonylureas, with better glycemic control and life quality [12,16]. In addition, clinicians had the opportunity to deepen the familial anamnesis, discovering the presence of cases of macrosomia and neonatal hypoglycemia which were initially attributed to the fact that they were born from a diabetic mother. HNF1A-MODY3 and HNF4A-MODY1 are phenotypically very similar, and it is likely that the clinician, given the higher frequency of HNF1A-MODY3, initially directed the patient to the analysis of the most probable MODY form [12,17,18]. 

In case 2, although, generally, the onset of HNF1B-MODY5 is in the adolescent age, however, hyperglycemia may be also evidenced in the neonatal period [13]; therefore, clinicians, particularly for the presence of renal malformations, directed the proband to *HNF1B* analysis. NGS evidenced instead a heterozygous mutation in the *GCK* gene that may be compatible with the neonatal insurgence of hyperglycemia, in accordance with another case reported in the literature of neonatal diabetes due to the heterozygote mutation of *GCK* [19]. In addition, the father (Figure 2, II:1) and the paternal grandfather of the patient (Figure 2, I:1) presented mild hyperglycemia and slightly altered HbA1c values (7.0% and 6.6%, respectively), compatible with GCK-MODY2 phenotype [20,21]. The patient was then also evaluated with a diagnostic flowchart, called 7-iF, based on seven clinical and laboratory characteristics, created in an attempt to improve the detection rate of GCK-MODY2 [22] and he effectively resulted positive to all criteria according to the molecular result. In this patient, NGS clarified the precise pathogenesis of his hyperglycemia, which was different from the first clinical suspicion. The diagnosis of GCK-MODY2 allowed him to avoid any unnecessary hypoglycemic treatment and made it possible to comfort the other affected relatives about the benign prognosis of their disease.

## 5. Conclusions

Monogenic diabetes in pediatrics has entered the routine of clinical differential diagnosis for a relatively short time and it is not always easy for the pediatrician to recognize the distinctive features that allow to accurately trace the specific gene involved in the pathogenesis. Furthermore, in order to make a precise diagnosis, it is often necessary to return to anamnestic information concerning the family which can often be overlooked at the time of diagnosis. In addition, diagnosis of pediatric diabetes could be also complicated by the simultaneous presence of different pathogenic mechanisms, autoimmune and monogenic [23]; therefore, it is necessary to make available to pediatricians and diabetologists all the most modern and sophisticated analyses to implement precision diabetology. 

The above-described clinical cases highlight the relevant role of NGS in precision medicine of monogenic diabetes, confirming the ability of this methodology to also show rare subtypes previously undiagnosed. The possibility to reach a “precise” diagnosis, in MD patients, allows clinicians to evaluate the severity and the progression of hyperglycemia, the risk of diabetic complications, the more appropriate therapy, and to perform familial genetic screening and counseling. 

## Data Availability

The authors confirm that the data supporting the findings of this study are available within the article or can be made available upon reasonable request.

## References

[1] Iafusco D., Zanfardino A., Bonfanti R., Rabbone I., Tinto N., Iafusco F., Meola S., Gicchino M.F., Ozen G., Casaburo F. (2020). Congenital Diabetes Mellitus. Minerva Pediatr..

[2] Bitterman O., Tinto N., Franzese A., Iafusco F., Festa C., Mozzillo E., Napoli A., Iafusco D. (2018). Glucokinase Deficit and Birthweight: Does Maternal Hyperglycemia Always Meet Fetal Needs?. Acta Diabetol..

[3] Delvecchio M., Mozzillo E., Salzano G., Iafusco D., Frontino G., Patera P.I., Rabbone I., Cherubini V., Grasso V., Tinto N. (2017). Monogenic Diabetes Accounts for 6.3% of Cases Referred to 15 Italian Pediatric Diabetes Centers During 2007 to 2012. J. Clin. Endocrinol. Metab..

[4] Donath X., Saint-Martin C., Dubois-Laforgue D., Rajasingham R., Mifsud F., Ciangura C., Timsit J., Bellanné-Chantelot C., Monogenic Diabetes Study Group of the Société Francophone du Diabète (2019). Next-Generation Sequencing Identifies Monogenic Diabetes in 16% of Patients with Late Adolescence/Adult-Onset Diabetes Selected on a Clinical Basis: A Cross-Sectional Analysis. BMC Med..

[5] Maturity-Onset Diabetes of the Young (MODY): How Many Cases Are We Missing?—PubMed. https://pubmed.ncbi.nlm.nih.gov/20499044/.

[6] Moran A., Pillay K., Becker D., Granados A., Hameed S., Acerini C.L. (2018). ISPAD Clinical Practice Consensus Guidelines 2018: Management of Cystic Fibrosis-Related Diabetes in Children and Adolescents. Pediatr. Diabetes.

[7] Iafusco F., Maione G., Mazzaccara C., Di Candia F., Mozzillo E., Franzese A., Tinto N. (2021). NGS Analysis Revealed Digenic Heterozygous GCK and HNF1A Variants in a Child with Mild Hyperglycemia: A Case Report. Diagnostics.

[8] Forlani G., Zucchini S., Di Rocco A., Di Luzio R., Scipione M., Marasco E., Romeo G., Marchesini G., Mantovani V. (2010). Double Heterozygous Mutations Involving Both HNF1A/MODY3 and HNF4A/MODY1 Genes: A Case Report. Diabetes Care.

[9] Richards S., Aziz N., Bale S., Bick D., Das S., Gastier-Foster J., Grody W.W., Hegde M., Lyon E., on behalf of the ACMG Laboratory Quality Assurance Committee (2015). Standards and Guidelines for the Interpretation of Sequence Variants: A Joint Consensus Recommendation of the American College of Medical Genetics and Genomics and the Association for Molecular Pathology. Genet. Med..

[10] Bingham C., Hattersley A.T. (2004). Renal Cysts and Diabetes Syndrome Resulting from Mutations in Hepatocyte Nuclear Factor-1beta. Nephrol. Dial. Transplant..

[11] Barretta F., Mirra B., Monda E., Caiazza M., Lombardo B., Tinto N., Scudiero O., Frisso G., Mazzaccara C. (2020). The Hidden Fragility in the Heart of the Athletes: A Review of Genetic Biomarkers. Int. J. Mol. Sci..

[12] Hattersley A.T., Patel K.A. (2017). Precision Diabetes: Learning from Monogenic Diabetes. Diabetologia.

[13] Zmysłowska A., Jakiel P., Gadzalska K., Majos A., Płoszaj T., Ben-Skowronek I., Deja G., Glowinska-Olszewska B., Jarosz-Chobot P., Klonowska B. (2022). Next- Generation Sequencing Is an Effective Method for Diagnosing Patients with Different Forms of Monogenic Diabetes. Diabetes Res. Clin. Pract..

[14] Iafusco F., Meola S., Pecoraro C., Mazzaccara C., Iafusco D., Tinto N. (2021). Prenatal Diagnosis of HNF1b Mutation Allows Recognition of Neonatal Dysglycemia. Acta Diabetol..

[15] Riddle M.C., Philipson L.H., Rich S.S., Carlsson A., Franks P.W., Greeley S.A.W., Nolan J.J., Pearson E.R., Zeitler P.S., Hattersley A.T. (2020). Monogenic Diabetes: From Genetic Insights to Population-Based Precision in Care. Reflections From a Diabetes Care Editors’ Expert Forum. Diabetes Care.

[16] Delvecchio M., Pastore C., Giordano P. (2020). Treatment Options for MODY Patients: A Systematic Review of Literature. Diabetes Ther..

[17] Iafusco F., De Sanctis P., Pirozzi D., Capone S., Lombardo B., Gambale A., Confetto S., Zanfardino A., Iolascon A., Pastore L. (2019). Molecular Diagnosis of MODY3 Permitted to Reveal a de Novo 12q24.31 Deletion and to Explain a Complex Phenotype in a Young Diabetic Patient. Clin. Chem. Lab. Med..

[18] Delvecchio M., Salzano G., Bonura C., Cauvin V., Cherubini V., d’Annunzio G., Franzese A., Giglio S., Grasso V., Graziani V. (2018). Can HbA1c Combined with Fasting Plasma Glucose Help to Assess Priority for GCK-MODY vs HNF1A-MODY Genetic Testing?. Acta Diabetol..

[19] Prisco F., Iafusco D., Franzese A., Sulli N., Barbetti F. (2000). MODY 2 Presenting as Neonatal Hyperglycaemia: A Need to Reshape the Definition of “Neonatal Diabetes”?. Diabetologia.

[20] Capuano M., Garcia-Herrero C.M., Tinto N., Carluccio C., Capobianco V., Coto I., Cola A., Iafusco D., Franzese A., Zagari A. (2012). Glucokinase (GCK) Mutations and Their Characterization in MODY2 Children of Southern Italy. PLoS ONE.

[21] Tinto N., Zagari A., Capuano M., De Simone A., Capobianco V., Daniele G., Giugliano M., Spadaro R., Franzese A., Sacchetti L. (2008). Glucokinase Gene Mutations: Structural and Genotype-Phenotype Analyses in MODY Children from South Italy. PLoS ONE.

[22] Pinelli M., Acquaviva F., Barbetti F., Caredda E., Cocozza S., Delvecchio M., Mozzillo E., Pirozzi D., Prisco F., Rabbone I. (2013). Identification of Candidate Children for Maturity-Onset Diabetes of the Young Type 2 (MODY2) Gene Testing: A Seven-Item Clinical Flowchart (7-IF). PLoS ONE.

[23] Maltoni G., Zucchini S., Scipione M., Mantovani V., Salardi S., Cicognani A. (2012). Onset of Type 1 Diabetes Mellitus in Two Patients with Maturity Onset Diabetes of the Young. Pediatr. Diabetes.

